# To the Editors of the Medical and Physical Journal

**Published:** 1809-03

**Authors:** James Hall

**Affiliations:** London


### *To the Editors of the Medical and Physical Journal.*

GENTLEMEN,

THERE is a disease affecting the hair of children, seemingly imported from the East and West Indies, that has of late become very prevalent, and baffles every means used for its extirpation. It usually makes its first appearance in a small round spot, that appears bare and scurfy, and that generally increases in size, while others break out in other parts of the head. Some call it a ring-worm, while others affirm that it is nothing but a scald-head. But does not a scald-head generally, or indeed always, turn to sores, which, it would appear, never happens in the above disorder?

Among the various remedies proposed for this disorder, the most common is sulphur and tar; but this is a nasty application, and so tedious, that it may be used for many months without effecting a cure. Gunpowder, Scotch snuff, infusions of tobacco, vinegar, and mushroom juice have all been tried, with different success; but nothing, as yet, appears to have been discovered, which can be depended on as a certain cure.

The matres familiæ, as well as many respectable people connected with academies, particularly in and about London, have had of late much trouble with this disorder, and are a good deal alarmed. From what I have seen of it, I am sometimes tempted to think that it is extremely infectious. At any rate, several of the bishops having laid their hands on some young person's heads, of the most genteel appearance, for confirmation, are careful in wiping them immediately after, and in washing them well, so soon as the business is over. If, therefore, any of your medical readers could make it convenient to turn his thoughts to this subject, and would have the goodness to give some information as to the predisposing cause, as well as the best way of preventing its spreading in large families, and of treating it, he will be entitled to the thanks of the public at large, and will oblige many of your readers, as well as him, who is, with due respect and good wishes,

Yours, &c.

JAMES HALL.

*London, August* 12, 808.

*Consider-*

